# Prevalence and subtypes of *Blastocystis* in wild rodents from three provinces in China

**DOI:** 10.3389/fvets.2024.1432741

**Published:** 2024-07-10

**Authors:** Zhen-Qiu Gao, Hai-Tao Wang, Qing-Yu Hou, Ya Qin, Xing Yang, Quan Zhao, He Ma

**Affiliations:** ^1^School of Pharmacy, Yancheng Teachers University, Yancheng, Jiangsu Province, China; ^2^College of Life Sciences, Changchun Sci-Tech University, Shuangyang, Jilin Province, China; ^3^College of Veterinary Medicine, Qingdao Agricultural University, Qingdao, Shandong Province, China; ^4^College of Animal Science and Technology, Jilin Agricultural University, Changchun, Jilin Province, China; ^5^Department of Medical Microbiology and Immunology, School of Basic Medicine, Dali University, Dali, Yunnan Province, China

**Keywords:** *Blastocystis*, wild rodents, prevalence, subtype, China

## Abstract

**Introduction:**

*Blastocystis* is one of the most critical intestinal protozoans in various hosts, including humans and mice. To determine the status of *Blastocystis* infection in wild rodents in China.

**Methods:**

A total of 344 faecal samples were collected from seven wild rodent species from three provinces, and the small subunit ribosomal RNA (*SSU* rRNA) genes of *Blastocystis* were amplified to determine their prevalence and subtypes.

**Results:**

Of the 344 samples, 54 (15.70%) were detected as *Blastocystis-*positive. The prevalence of *Blastocystis* was 26.14% (40/153), 7.95% (7/88), and 6.80% (7/103) in wild rodents from Hunan Province, Yunnan Province, and Guangxi Province, respectively. The prevalence of *Blastocystis* in different wild rodent species varied from 0.00% (0/13) in *Mus musculus* to 40.00% (2/5) in *Rattus rattus sladeni*. The prevalence of *Blastocystis* in samples from the lake beach area (27.40%, 40/146) was significantly higher than in those from the mountain (6.80%, 7/103) and field regions (7.37%, 7/95). The prevalence in different seasons was 26.14% in summer (40/153), 7.95% in autumn (7/88), and 6.80% in winter (7/103). Moreover, a total of two *Blastocystis* subtypes were identified in the investigated wild rodents, including ST4 and ST5.

**Discussion:**

The present study discovered the existence of *Blastocystis* infection in *Rattus favipectus*, *Microtus fortis*, *Apodemus agrarius*, *Bandicota indica*, *Rattus rattus sladeni*, and *Rattus losea*, expanding the host range of this parasite. The findings also demonstrate that wild rodents may be an important potential infection source for *Blastocystis* infection in humans and other animals.

## 1 Introduction

Wild rodents are widely distributed worldwide. They are one of the most pivotal reservoir hosts for many pathogens, including bacteria (1), viruses (2), and parasites (3), and can transmit these pathogens between animals and humans. Thus, an increasing amount of research is being conducted regarding the epidemiology of zoonotic pathogens in wild rodents. *Blastocystis* is one of the most critical zoonotic pathogens; it can infect a variety of hosts, including humans (4) and wild mice (5). *Blastocystis* infection mainly occurs through the faecal-oral route, such as the ingestion of *Blastocystis* cysts-contaminated food or water (6), and is often associated with irritable bowel syndrome (IBS) (7), cutaneous allergic disorders (8), nausea, and diarrhoea (9). However, recently, some research has also suggested that some subtypes of *Blastocystis* may also be beneficial for health (10, 11). Hence, investigating the distribution of subtypes of *Blastocystis* is of particular importance for prevention and control of *Blastocystis* infection.

To date, a total of 42 *Blastocystis* subtypes (STs) have been identified based on the small subunit ribosomal RNA (*SSU* rRNA) gene (12, 13). Of these, more than 40 *Blastocystis* subtypes have previously been found in humans and animals (14). ST1-ST10, ST12, ST14, ST16 ST23, ST35, and ST41 have been found in humans with a predominance of ST1-ST4 (15), and all other subtypes have only been documented in animals (14). ST1-ST5, ST8, ST13, ST14, and ST17 have been identified in wild rodents, which suggests a potential risk of the zoonotic transmission of *Blastocystis* from wild rodents to humans (16). More importantly, ST1-3 and ST8 have not only been found in humans and wild rodents, but also in water (16), suggesting a higher risk of transmission of *Blastocystis* between humans and rodents through water sources.

In view of the above, the investigation of the prevalence and distribution of *Blastocystis* in wild rodents is important to prevent and control *Blastocystis* infection in different hosts. While a few studies have been published on *Blastocystis* infection in some rodents, these studies did not specifically examine the prevalence of subtypes or conduct subtype distribution analysis of *Blastocystis* in *Rattus favipectus*, *Microtus fortis*, *Apodemus agrarius*, *Bandicota indica*, *Rattus rattus sladeni*, and *Rattus losea*. Considering the lack of current data related to the occurrence of *Blastocystis* and the prevalence of subtypes in the above rodent species, in the present study, a total of 344 wild rodents were collected from Hunan Province, Yunnan Province, and Guangxi Province, China, and the *SSU* rRNA genes were amplified to investigate the prevalence of *Blastocystis.*

## 2 Materials and methods

### 2.1 Specimen collection and preparation

A total of 344 wild rodents (*n* = 139, *Microtus fortis*; *n* = 31, *Rattus norvegicus*; *n* = 14, *Apodemus agrarius*; *n* = 39, *Bandicota indica*; *n* = 5, *Rattus rattus sladeni*; *n* = 39, *Rattus flavipectus*; *n* = 41, *Rattus losea*; *n* = 23, *Niviventer lotipes*; *n* = 13, *Mus musculus*) were randomly collected from Yunnan Province (*n* = 88, 21°8′ ~ 29°15′ N, 97°31′ ~ 106°11′ E), Hunan Province (*n* = 153, 24°38′ ~ 30°08′ N, 108°47′ ~ 114°15′ E), and Guangxi Province (*n* = 103, 20°54′ ~ 26°24′ N, 104°28′ ~ 112°04′ E), China, between September 2023 and February 2024. Information including gender, sampling times, seasons, regions, and species were recorded. All wild rodents were captured using mouse traps, and faecal samples were collected from the rectum of each rodent. Then, faecal samples were placed into a box with dry ice and sent to the laboratory. The DNA of each faecal sample was extracted using a Stool DNA kit (OMEGA, United States), according to the manufacturer’s instructions, and kept at −20°C until analysis via polymerase chain reaction (PCR).

### 2.2 PCR amplification, sequencing and phylogenetic analyses

The *SSU* rRNA genes of *Blastocystis* were amplified to determine their subtypes (6). Using the primers RD5 (5′- ATCTGGTTGATCCTGCCAGT-3′) and BhRDr (5′-GAGCTTTTTAACTGCAACAACG-3′), the *SSU* rRNA gene was amplified using PCR at a region of around 600 bp. Both the positive control (sequenced isolates of *Blastocystis*) and negative control (sterile distilled water) were also amplified in each test. The electrophoresis of 6 μL PCR products was performed on 1.0% agarose gel in TBE. All of the target products were detected under UV light and sequenced based on bidirectional sequencing at the General Biol. Company in Anhui, China. Then, the sequences were blasted with known reference sequences available in GenBank. The neighbour-joining (NJ) method (Kimura 2-parameter model and 1,000 replicates) was used to analyse the phylogenetic relationships of these *Blastocystis* based on Mega 5.0.^1^

### 2.3 Statistical analysis

The chi-square test in SAS (Statistical Analysis System, Version 9.0) was used to calculate the difference between the prevalence of *Blastocystis* (*y*) and different factors, including seasons (*x1*), species (*x2*), gender (*x3*), regions (*x4*), and environment (*x5*). The difference was considered as statistically significant if *p* < 0.05. Moreover, the chi-square test in SPSS (IBM Corp., Armonk, NY, United States) was used to calculate the odds ratios (ORs) and their 95% confidence intervals (95% CIs).

## 3 Results

A total of 344 wild rodents were collected in the present study. Of these, 61 were identified as *Blastocystis-*positive through the PCR of *SSU* rRNA genes of *Blastocystis*. The total *Blastocystis* prevalence was 15.70% (54/344), with 26.14% (40/153) in Hunan, 7.95% (7/88) in Yunnan, and 6.80% (7/103) in Guangxi (Table 1). The prevalence of *Blastocystis* from the lake beach area (27.40%, 40/146) was significantly higher than that from the mountain (6.80%, 7/103) and field regions (7.37%, 7/95). The prevalence varied from 6.80% in winter (7/103) to 26.14% in summer (40/153). The prevalence in the wild rodents of according to gender was 16.24% for males and 14.97% for females.

**Table 1:** Factors associated with prevalence of *Blastocystis* in wild rodents in China.

Factor	Category	No. tested	No. positive	% (95%CI)	*p*-value	OR (95%CI)
Region	Guangxi Province	103	7	6.80 (2.61–12.59)	< 0.0001	Reference
	Yunnan Province	88	7	7.95 (3.07–14.67)		1.19 (0.40–3.52)
	Hunan Province	153	40	26.14 (19.46–33.42)		4.85 (2.08–11.33)
Species	*Bandicota indica*	39	1	2.56 (4.00–25.00)	< 0.0001	Reference
	*Microtus fortis*	139	39	28.06 (21.00–36.00)		14.82 (1.97–111.69)
	*Rattus norvegicus*	31	2	6.45 (0.00–18.00)		2.62 (0.23–30.32)
	*Rattus rattus sladeni*	5	2	40.00 (13.00–98.00)		25.33 (1.75–366.83)
	*Rattus flavipectus*	39	3	7.69 (1.01–18.58)		3.17 (0.32–31.86)
	*Rattus losea*	41	6	14.63 (5.20–27.34)		6.51 (0.74–56.84)
	*Apodemus agrarius*	14	1	7.14 (0.00–28.00)		2.92 (0.17–50.15)
	*Niviventer lotipes*	23	0	0.00 (−)		-
	*Mus musculus*	13	0	0.00 (−)		-
Gender	Female	147	22	14.97 (9.61–21.23)	0.7601	Reference
	Male	197	32	16.24 (11.40–21.75)		1.10 (0.61–1.99)
Environment	Mountain	103	7	6.80 (2.61–12.59)	< 0.0001	Reference
	Lakebeach	146	40	27.40 (20.44–34.94)		5.18 (2.21–12.10)
	Field	95	7	7.37 (2.83–13.62)		1.10 (0.37–3.23)
Total		344	54	15.70 (12.05–19.73)		

The effect of regions, species, seasons, environment and gender regarding *Blastocystis* infection was analysed using forward stepwise logistic regression analysis based on Fisher’s scoring technique. Only region was included as a variable in final model, which has a close association with *Blastocystis* infection. The equation is described as follows: *y* = 0.9097*×4* + 1.0973. Region had strongly effects on the *Blastocystis* infection in the wild rodents, for which the OR was 2.48 (95% CI 1.61–3.82). Wild rodents from Yunnan (OR 1.19, 95% CI 0.40–3.52) and Hunan (OR 4.85, 95% CI 2.08–11.33) were seen to be more susceptible than those from Guangxi (Table 1).

A total of two subtypes were identified in the present study, namely, ST4 and ST5. Of these, ST4 was the predominant *Blastocystis* subtype, being found in seven rodent species (*n* = 39, *Microtus fortis*; *n* = 2, *Rattus norvegicus*; *n* = 1, *Apodemus agrarius*; *n* = 1, *Bandicota indica*; n = 2, *Rattus rattus sladeni*; *n* = 2, *Rattus flavipectus*; *n* = 6, *Rattus losea*) in all three provinces (*n* = 40, Hunan Province; *n* = 6, Yunnan Province; *n* = 7, Guangxi Province), followed by ST5, which was only found in *Rattus flavipectus* (*n* = 1) collected in winter in Yunnan Province. However, ST4 and ST5 did not appear in the same rodent. ST4 was found in the lake beach, mountain, and field areas, whereas ST5 was only found in the mountain area (Table 2).

**Table 2:** Distribution of *Blastocystis* subtypes.

Factor	Category	No. positive/No. tested (%)	Subtypes (No.)
Region	Yunnan Province	7/88 (7.95)	ST4 (*n* = 6); ST5 (*n* = 1)
	Hunan Province	40/153 (26.14)	ST4 (*n* = 40)
	Guangxi Province	7/103 (6.80)	ST4 (*n* = 7)
Species	*Rattus flavipectus*	3/39 (7.69)	ST4 (*n* = 2); ST5 (*n* = 1)
	*Microtus fortis*	39/139 (28.06)	ST4 (*n* = 39)
	*Rattus norvegicus*	2/31 (6.45)	ST4 (*n* = 2)
	*Apodemus agrarius*	1/14 (7.14)	ST4 (*n* = 1)
	*Bandicota indica*	1/39 (2.56)	ST4 (*n* = 1)
	*Rattus rattus sladeni*	2/5 (40.00)	ST4 (*n* = 2)
	*Rattus losea*	6/41 (14.63)	ST4 (*n* = 6)
	*Niviventer lotipes*	0/23 (0.00)	–
	*Mus musculus*	0/13 (0.00)	–
Season	Autumn	7/88 (7.95)	ST4 (*n* = 6); ST5 (*n* = 1)
	Summer	40/153 (26.14)	ST4 (*n* = 40)
	Winter	7/103 (6.80)	ST4 (*n* = 7)
Gender	Male	32/197 (16.24)	ST4 (*n* = 31); ST5 (*n* = 1)
	Female	22/147 (14.97)	ST4 (*n* = 22)
Environment	Field	7/95 (7.37)	ST4 (*n* = 6); ST5 (*n* = 1)
	Lakebeach	40/146 (27.40)	ST4 (*n* = 40)
	Mountain	7/103 (6.80)	ST4 (*n* = 7)
Totol		54/344 (15.70)	ST4 (*n* = 53); ST5 (*n* = 1)

**Figure 1:**
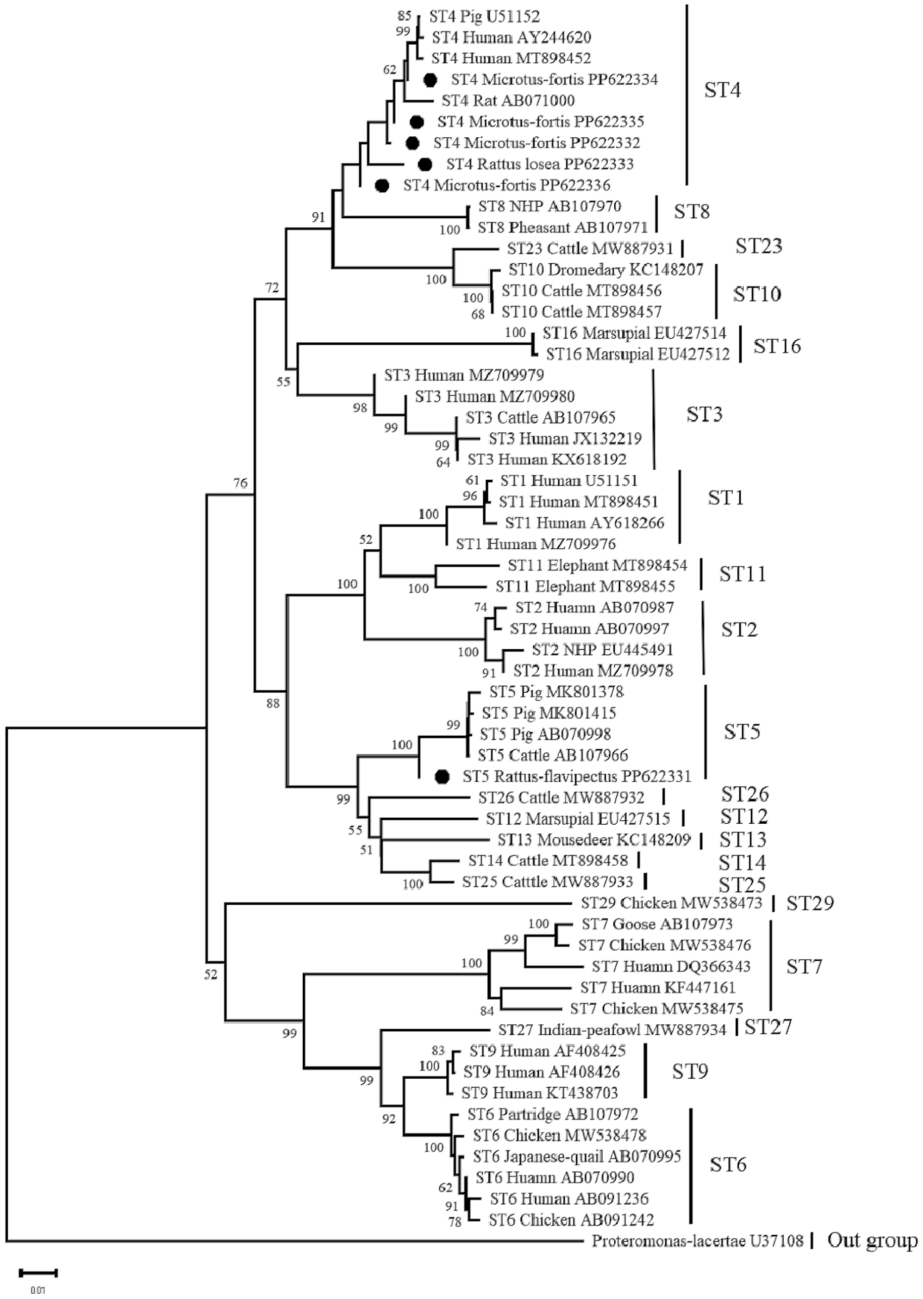
Neighbour-joining (NJ) phylogenetic analyses of *Blastocystis* based on the *SSU* rRNA genes. Bootstrap values more than 50% are shown. The *Blastocystis* isolates detected in present study are indicated by black circles.

A total of six representative sequences were obtained from the 54 *Blastocystis* isolates in the present study. The ST4 sequences (PP622334, PP622335, PP622332, and PP622333) showed 100% homology with isolates from humans (MN836841, MH784408) reported in GenBank. The ST4 sequence (PP622336) in this study was also identical to sequences from other rodents such as rodents in Mexico (MK251246) and *Rattus exulans* in Indonesia (MH127488). On the other hand, the ST5 sequence (PP622331) was 100% homologous to isolates from pigs (MN526819, MK801414, and KY610202) reported in GenBank (Figure 1).

## 4 Discussion

In the present study, 54 out of 344 wild rodents were *Blastocystis*-positive based on a PCR of *SSU* rRNA genes. The overall prevalence was 15.70%, which was lower than that observed previously in rodents in Spain (83.5%) (17), Malaysia (45.9%) (18), Japan (44.4%) (19), England (43.5%) (20), and Ecuador (35.4%) (21); close to that in Indonesia (16.4%) (22), Iran (15.7%) (5); and higher than that previously reported in Brazil (8.7%) (23) and Mexico (13.3%) (24). *Blastocystis* infection in rodents has also been surveyed in other cities in China, with infection rates ranging from 0.0 to 27.3%, such as Sichuan, Hunan, Heilongjiang, Hainan, and Hubei, where the infection rates were 8.4, 4.6, 3.7, 2.2, and 30.4%, respectively (25–31). The different prevalence of *Blastocystis* in different studies may be due to the different sampling times, different susceptibility in different animals, and different pollution levels of the environment. Importantly, *Blastocystis* is widely found in different regions, suggesting that these regions are commonly contaminated by *Blastocystis* cysts, which should be paid more attention to during the prevention and control of the disease.

In the present study, the difference in the prevalence of *Blastocystis* in the summer, autumn, and winter was significant, indicating that the extent of parasitic infection varied significantly between seasons. Similar to a previous Japanese study, the rodents had lower rates of *Blastocystis* infection in winter (19). Several studies have shown that temperature and precipitation are positively correlated with parasites infection intensity, possibly because the higher temperatures and precipitation in summer and wet seasons are conducive to the growth and transmission of parasites. Moreover, the activity of wild rodents in winter was less than that in summer, which reduced the contact between wild rodents and other animals and water sources, thereby reducing the transmission of *Blastocystis*. Although the infection rate of *Blastocystis* was higher in summer than in winter, it was detected in summer, winter, and autumn, indicating that *Blastocystis* could be transmitted all year round and is a zoonotic parasite that requires significant attention. *Blastocystis* was reportedly found in water sources in 15 countries around the world, and the infection rate of some water sources, including fountain water, rainwater, rivers, stored water, and irrigation water, was as high as 100% (32). Studies also showed that drinking unboiled water was found to correlate with a high prevalence of *Blastocystis* infections in China (33, 34). *Blastocystis* is also one of the water-related pathogens in the WHO’s drinking water quality publications, which indicates the public health significance of this parasite (35, 36). Therefore, hosts can acquire *Blastocystis* infection through the contaminated water, which is also the most common route of infection. Noteworthy is that *Blastocystis* cysts can survive for 19–30 days in water at a temperature of 25°C and for 2 months at 4°C (37). This tendency is consistent with the results of this study, as more *Blastocystis* were detected near water sources. The prevalence of *Blastocystis* from lake beach areas (27.40%, 40/146) was significantly higher (*p* < 0.0001) than those from the mountain (6.80%, 7/103) and field regions (7.37%, 7/95); this suggests that access to safe drinking water to avoid *Blastocystis* infection is critical to public health.

In this study, the positive rate was 14.97% (22/147) in females, and 16.24% (32/197) in males. There was no significant difference in the positive rate between different gender groups (*p* > 0.05), which suggests that the infection rates are not affected by gender; similar results were also observed in a Japanese survey (19), where *Blastocystis* was found in seven species of wild rodents, including *Bandicota indica*, *Microtus fortis*, *Rattus norvegicus*, *Rattus rattus sladeni*, *Rattus flavipectus*, *Rattus losea*, and *Apodemus agrarius*, indicating that this zoonotic parasite is widely distributed in wild rodents. It is worth noting that *Blastocystis* infection was discovered for the first time in *Bandicota indica*, *Microtus fortis*, *Rattus flavipectus*, *Rattus rattus sladeni*, and *Apodemus agrarius.*

To date, a total of 42 *Blastocystis* subtypes (STs) have been identified through the utilisation of DNA-based techniques and sequence analysis of the small subunit ribosomal RNA (*SSU* rRNA) gene (12, 13). A large number of studies have reported that ST4 is the dominant subtype of wild rodents and is distributed in different species of rodents and different countries, such as China (25), Indonesia (22), Japan (19), Malaysia (18), Iran (38), and the United Kingdom (20). ST4 is also one of the most common subtypes in humans (10). In addition, ST4 has a peculiar geographical distribution and is the most influenced by geography and lifestyle (39). ST4 was originally isolated from a Wistar rat (40). Subsequently, ST4 was detected in wild rodents, cows, goats, pigs, and other wild mammals worldwide, and especially in the water sources that humans and animals are exposed to Liu et al. and Shams et al. (27, 41). These findings indicated that ST4 has a wide host range, infected wild animals can contaminate drinking water, and the consumption of contaminated water or contact with contaminated surface water may expose humans to *Blastocystis*. In the present study, ST4 was found in seven wild rodent species in all regions. The results demonstrated that ST4 is one of the most critical zoonotic subtypes of *Blastocystis* and requires more attention in future research.

Hoofed animals are the natural hosts of ST5, including cattle (42), pigs (43), sheep (44), and camels (45). Some studies have shown that ST5 is the predominant subtype in pigs, and has also been identified in humans working in commercial intensive pig farms (43), suggesting that close contact or exposure to infected animals may be an important route of infection for ST5 (46). Zoonotic ST5 has only been detected sporadically in rodents; for instance, *Blastocystis* ST5 was identified in *Hydrochoerus hydrochaeris* in France (47), in *Clethrionomys glareolus* in the United Kingdom (48), in *Rattus norvegicus* in Malaysia (18), and in *Myocastor coypus*, *Rhizomys sinensis*, and *Callosciurus erythraeus* in China (27, 28, 49). ST5 has also been found in rivers and lakes. In the present study, ST5 was only identified in *Rattus flavipectus* from the mountain regions in Yunnan. These findings suggest that although ST5 is rare in rodents, it can still be transmitted between humans and animals, posing a potential zoonotic risk.

Although this study provided valuable evidence of *Blastocystis* infections in rodents, there are several limitations that need to be acknowledged. The rodent samples obtained in this study encompassed a limited geographical area, which is insufficient for a comprehensive understanding of the prevalence of *Blastocystis* infection in rodents in China. Furthermore, the number of positive samples in this study was limited, which may have resulted in an inadequate representation of *Blastocystis* genetic diversity. To gain a more comprehensive understanding of the prevalence of *Blastocystis*, it is necessary to expand the sample size and collection area in follow-up studies.

## 5 Conclusion

The findings of this study demonstrated that *Blastocystis* infection in wild rodents is a frequently occurrence in China. The present study also discovered the existence of *Blastocystis* infection in *Rattus favipectus*, *Microtus fortis*, *Apodemus agrarius*, *Bandicota indica*, *Rattus rattus sladeni*, and *Rattus losea*, thereby broadening the host range of this parasite. Region, species, season, and environment had strong effects on *Blastocystis* infection in the investigated wild rodents. Crucially, ST4 and ST5, previously found in humans, were also found in this study, which suggests that wild rodents may be an important potential sources of human infections. Our study provided reliable data for future studies on *Blastocystis* subtype distribution in rodents and *Blastocystis* infection control in wild animals in China.

## Data availability statement

The representative gene sequence was submitted to GenBank (Accession nos. PP622331–PP622336).

## Ethics statement

The animal study was approved by the Animal Ethics Committee of Yancheng Teachers University. The study was conducted in accordance with the local legislation and institutional requirements.

## Author contributions

Z-QG: Methodology, Software, Writing – original draft. H-TW: Methodology, Software, Writing – original draft. Q-YH: Data curation, Writing – review & editing. YQ: Data curation, Resources, Visualization, Writing – review & editing. XY: Data curation, Visualization, Writing – review & editing. QZ: Conceptualization, Resources, Supervision, Writing – review & editing. HM: Conceptualization, Resources, Writing – review & editing.

## Footnotes

^1^ http://www.megasoftware.net/
